# Supplementary material for: The origin and maintenance of supergenes contributing to ecological adaptation in Atlantic herring
Source: Nat Commun. 2024 Oct 23;15:9136. doi: 10.1038/s41467-024-53079-7 (PMC11499932; doi:10.1038/s41467-024-53079-7)
Supplement: Supplementary file 1 — Supplementary Information [file 41467_2024_53079_MOESM1_ESM.pdf]

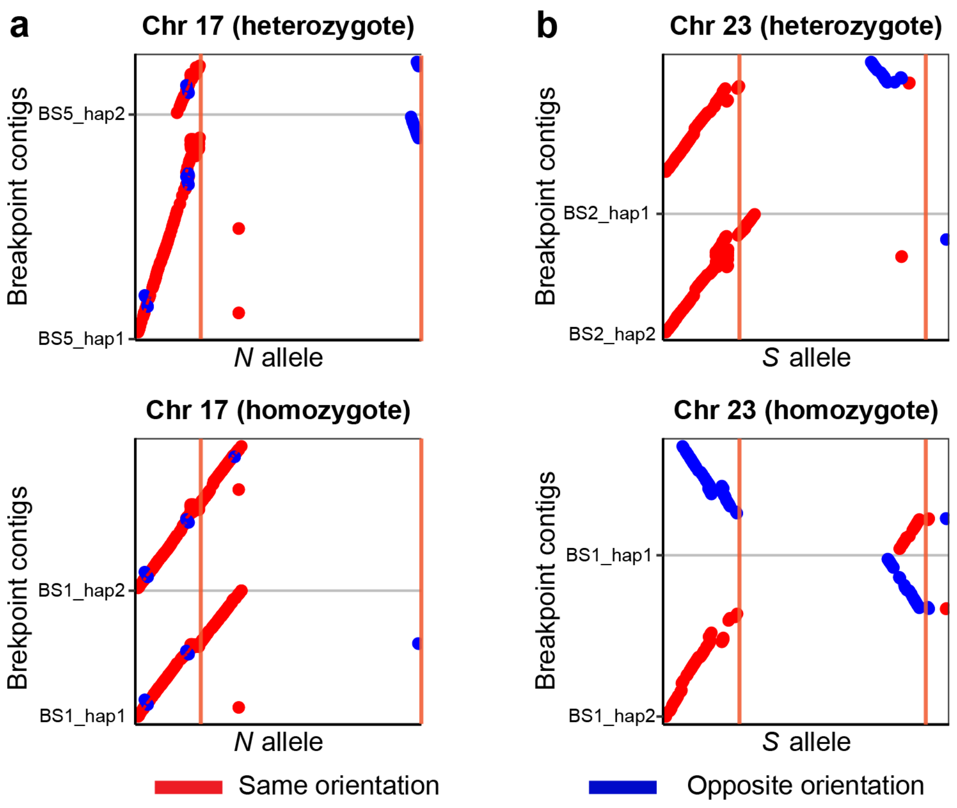

Supplementary Fig. 1. **Dot plot of the PacBio proximal breakpoint contigs from hap1 and hap2 assemblies (Y-axis) for two heterozygotes and two homozygotes compared with the inversion alleles in the reference assembly (X-axis).** Vertical pink lines are the inversion breakpoints and the horizontal line divides the data from the two haplotypes. **a** Chr17. BS5 is heterozygous for Chr17 inversion, where BS5_hap1 assembly has *S* haplotype and BS5_hap2 assembly has *N* haplotype. The homozygote (*NN*) sample (BS1) is used as a control. **b** Chr23. BS2 is heterozygous for Chr23 inversion, where BS2_hap1 assembly has *N* haplotype and BS2_hap2 assembly has *S* haplotype. The homozygote sample (BS1) is used as a control with *NN* arrangement. The sequence lengths for Chr17 *N* allele and Chr23 *S* allele are 2.8 Mb and 3.0 Mb, respectively. This analysis was performed for all individuals subjected to PacBio sequencing.

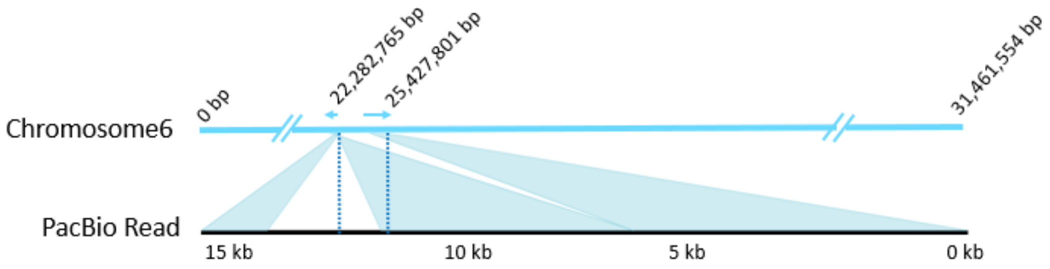

Supplementary Fig. 2. Single PacBio read spanning proximal and distal breakpoint of Chr6 inversion from the CS2 sample on the reference sequence. The inversion breakpoints are shown in dotted lines.

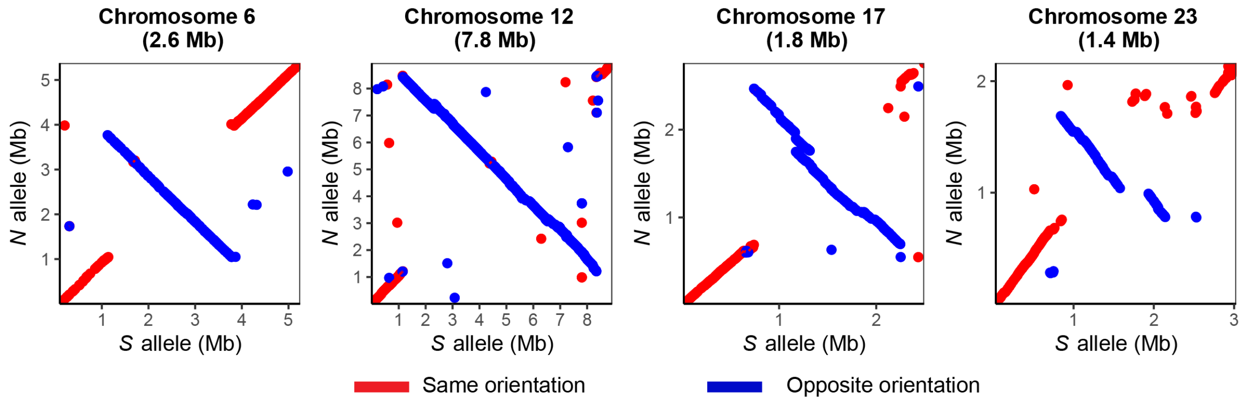

Supplementary Fig. 3. Sequence alignment of *N* and *S* alleles for all four inversions. *N* and *S* alleles are represented by the CS10_hap1 and BS3_hap2 inversion scaffolds, respectively.

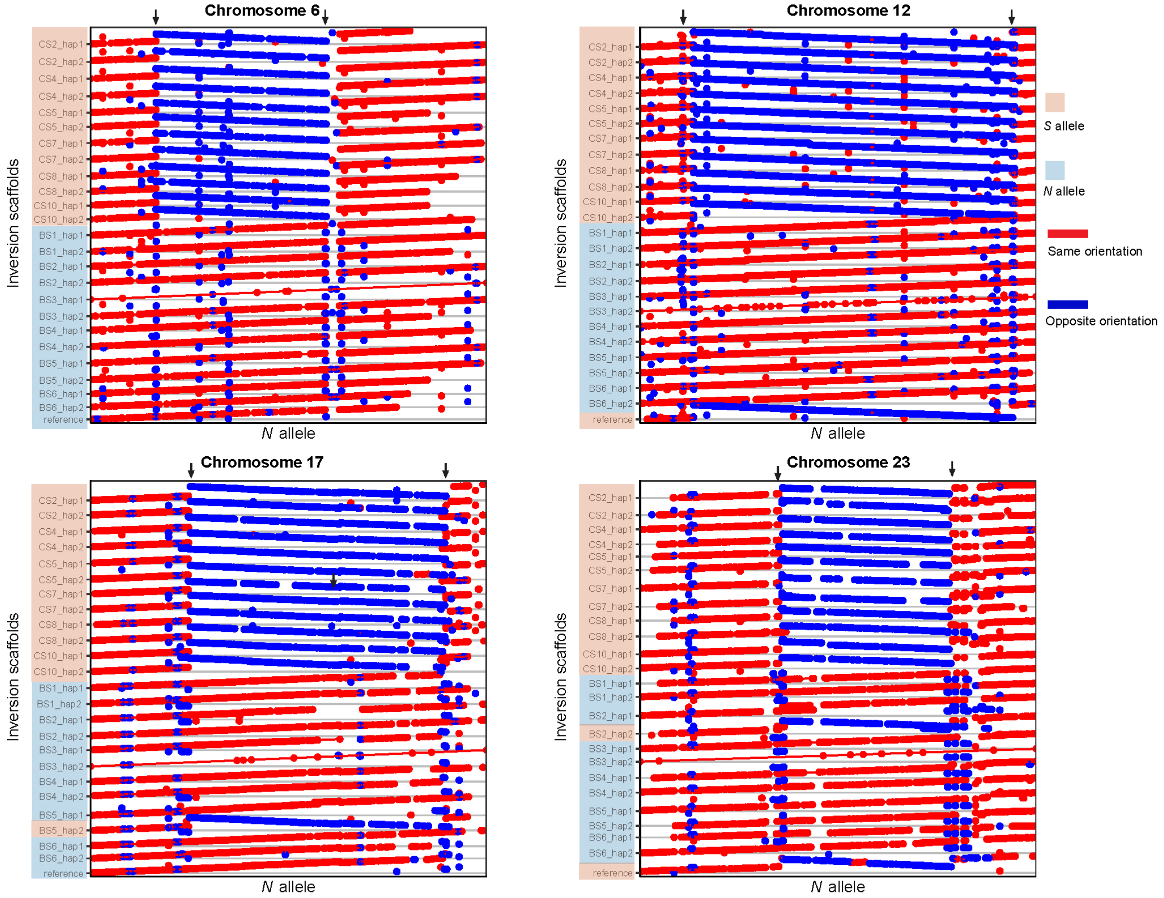

Supplementary Fig. 4. **Dot plots of inversion alleles.** Y-axis has all inversion alleles assembled in this study from PacBio assemblies and the reference assembly. X-axis has a reference inversion *N* allele constructed using CS10_hap1 assembly. Inverted duplications at the breakpoint of chromosome 6, 12, and 17 inversions are indicated in the figure by black arrows above the dot plots. Red color indicates alignment in the same orientation and blue color indicates alignment in the opposite orientation. The sequence lengths for chromosome 6, 12, 17, and 23 inversion scaffolds are nearly 5.0 Mb, 9.0 Mb, 2.8 Mb, and 3.0 Mb, respectively.

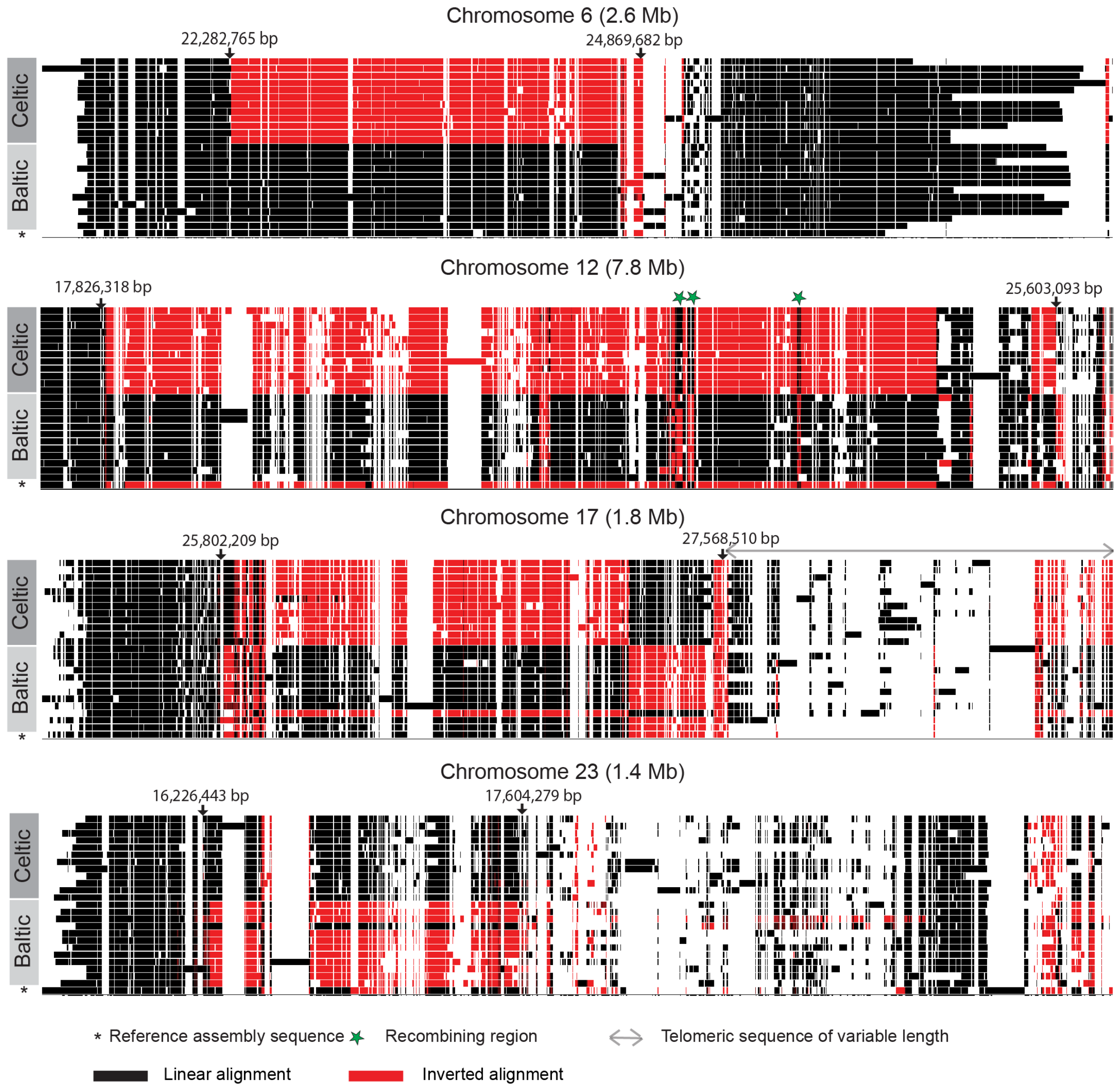

Supplementary Fig. 5. **Pangenome graphs using 25 sequences (24 inversion scaffolds and one reference assembly) for inversions on chromosomes 6, 12, 17, and 23.** Top 12 sequences are from Celtic Sea samples and the next 12 sequences are from Baltic Sea samples. The last sequence indicated with an asterisk is from the reference assembly. Black and red colors represent opposite orientations of the sequence hence, the vertical boundary of black and red are the inversion breakpoints, also indicated in bp at the top. Possible recombining regions on Chr12 inversions are indicated by green stars at the top of its genome graph. Variable telomeric sequence (0-300 kb in range) outside the breakpoint on Chr17 is indicated by grey line. The sequence lengths for chromosome 6, 12, 17, and 23 pangenome graphs are nearly 5.0 Mb, 9.0 Mb, 2.5 Mb, and 3.0 Mb, respectively.

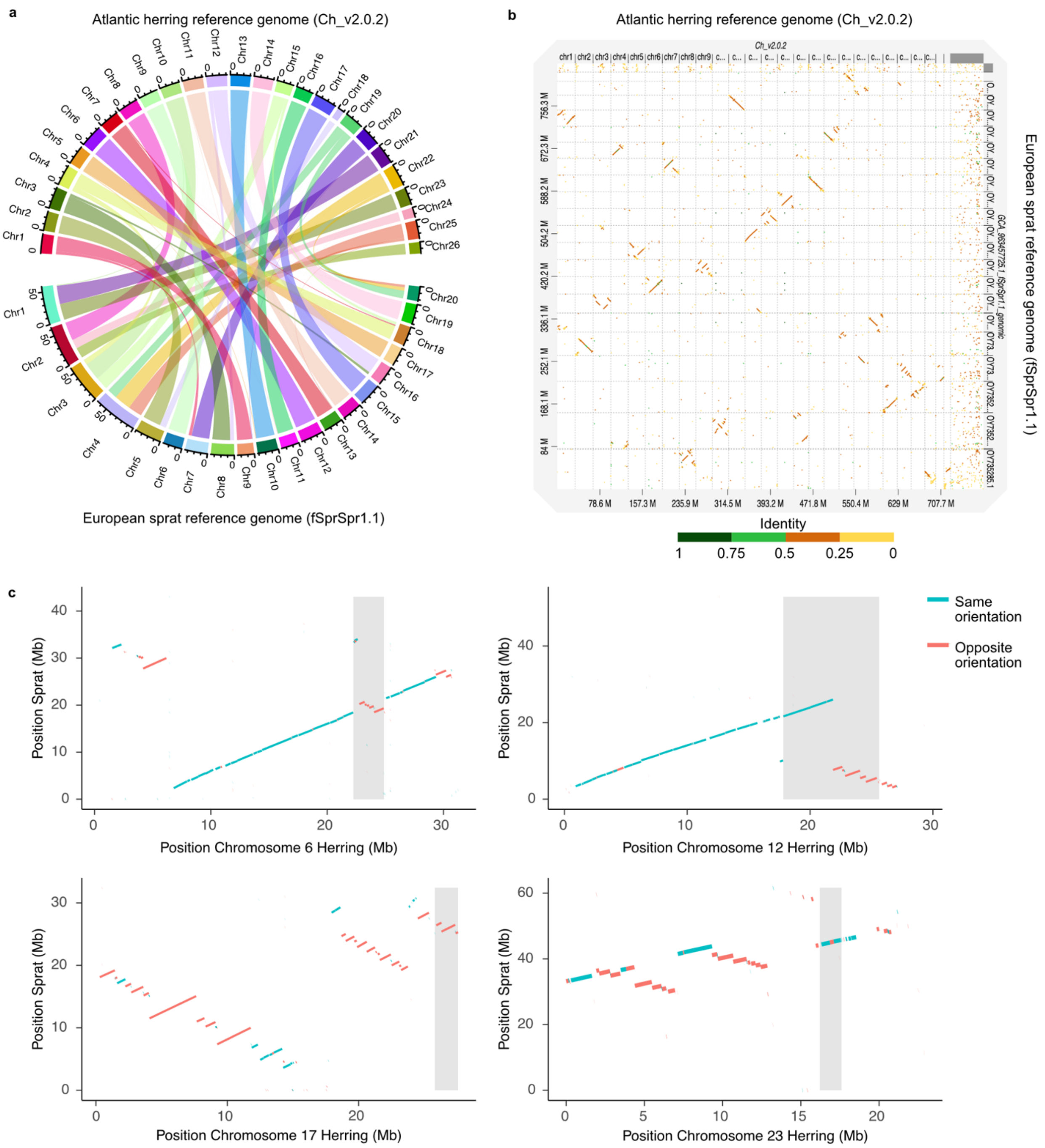

Supplementary Fig. 6. Sequence comparison of Atlantic herring and European sprat genome assemblies and herring inversions. a Synteny between the Atlantic herring and the European sprat reference genomes as proportion of aligned regions. **b** D-GENIES dot plot of Atlantic herring and European sprat reference genomes showing alignment blocks longer than 10 kb and with identity higher than 0.25. **c** Dot plots for the four Atlantic herring chromosomes containing inversions (chromosomes 6, 12, 17, and 23).

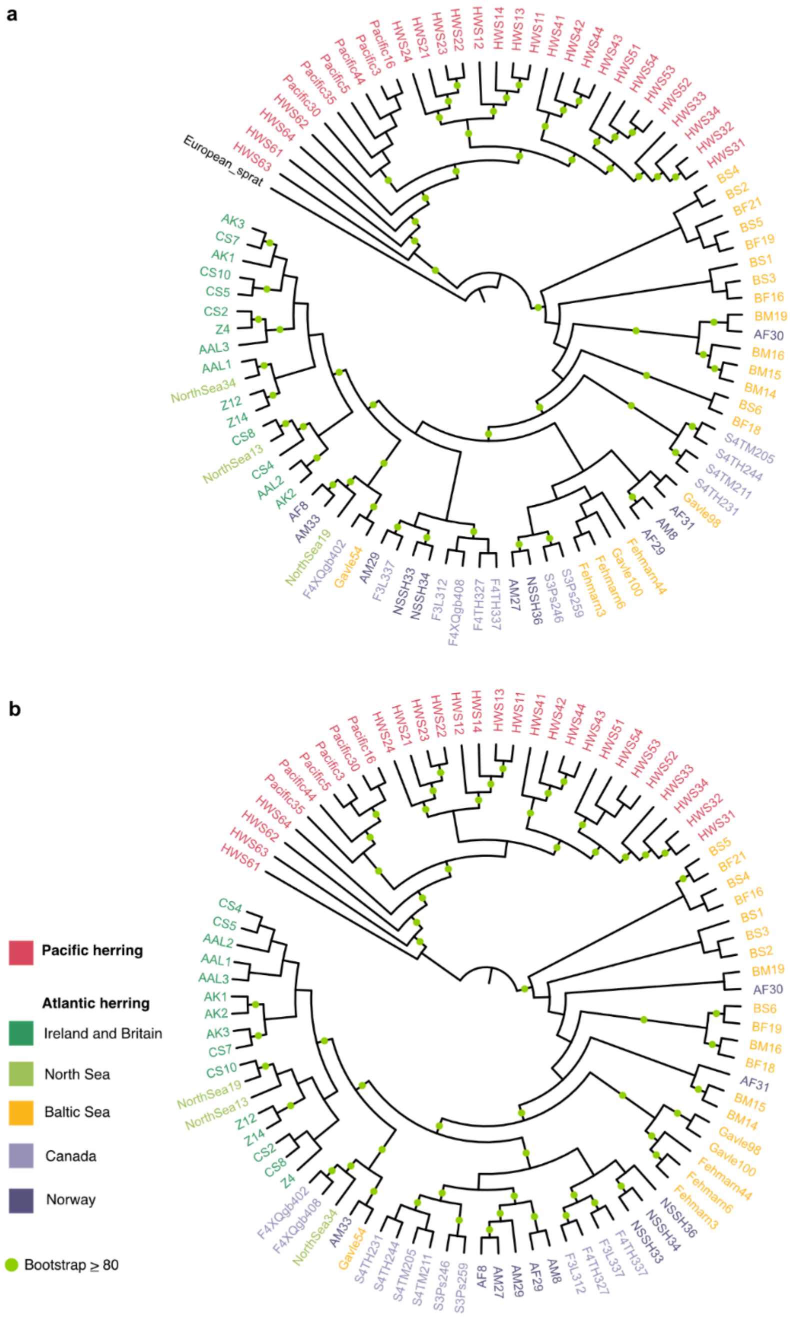

Supplementary Fig. 7. The evolutionary history of Atlantic and Pacific herring. Maximum likelihood trees (branch lengths were ignored to facilitate visualization of relationships among individuals) that are rooted with **a** European sprat or **b** at the branch that connects Pacific and Atlantic herring, following the topology of the tree in **a**. A concatenated alignment of 15,471 genes (~114 Mb) was used to produce tree in panel **a**, and a genome-wide alignment of 345,966,161 positions with no missing data was used to produce the tree in panel **b**.

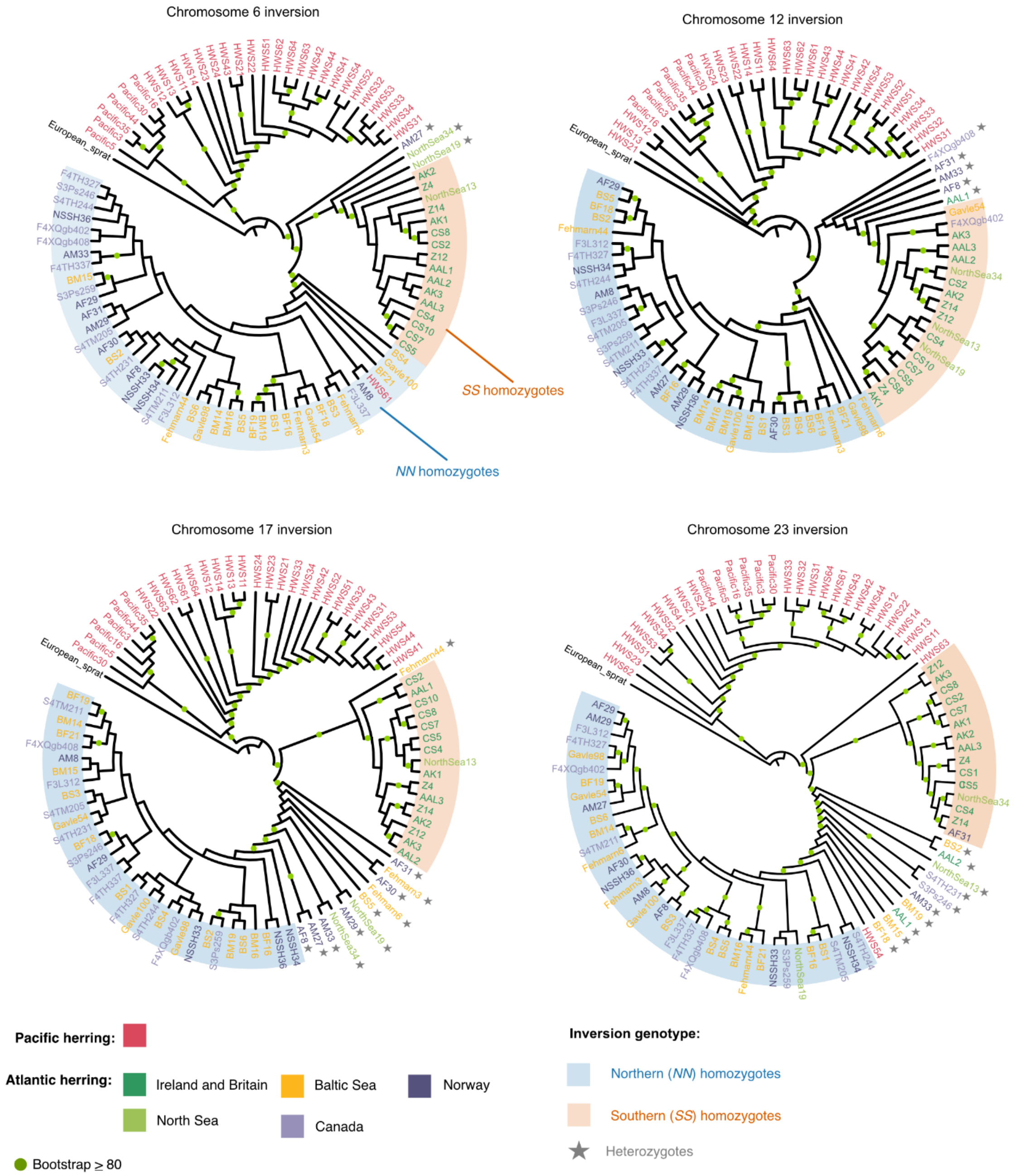

Supplementary Fig. 8. The evolutionary history of Atlantic herring chromosomal inversions. Maximum likelihood cladograms (branch lengths of maximum likelihood trees were ignored to facilitate visualization of relationships among individuals) of concatenated alignments of chromosome 6, 12, 17 and 23 inversion regions, including the European sprat as an outgroup. Individuals are color coded by species or Atlantic herring population. Shades behind individuals indicate their inversion genotype according to Supplementary Fig. 2, and heterozygotes are indicated with a star.

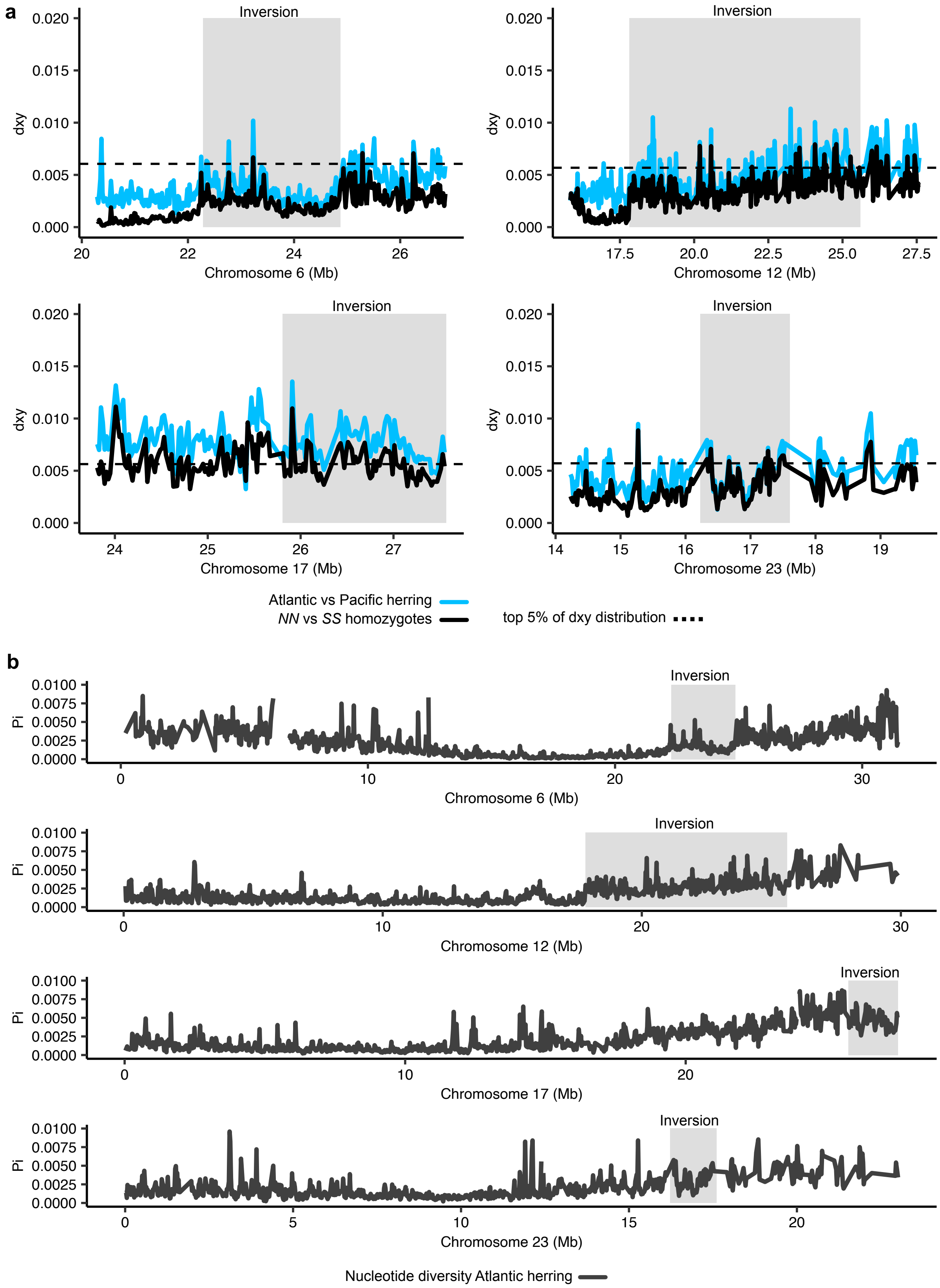
a
Inversion
dxy
0.020
0.015
0.010
0.005
0.000
Chromosome 6 (Mb)
Chromosome 12 (Mb)
Chromosome 17 (Mb)
Chromosome 23 (Mb)
Atlantic vs Pacific herring
NN vs SS homozygotes
top 5% of dxy distribution
b
Pi
0.0100
0.0075
0.0050
0.0025
0.0000
Nucleotide diversity Atlantic herring

Supplementary Fig. 9. Divergence and diversity in and around inversion regions in Atlantic herring. a Divergence (d_{xy}) between North and South homozygotes for inversion alleles (black line) compared to divergence between Atlantic (n = 61) and Pacific (n = 30) herring (blue line). Number of North homozygous for *N* alleles is $n_{chr6} = 20$, $n_{chr12} = 19$, $n_{chr17} = 16$ and $n_{chr23} = 16$, and number of South homozygous for *S* alleles is $n_{chr6} = 15$, $n_{chr12} = 15$, $n_{chr17} = 15$ and $n_{chr23} = 13$. The dashed line represents the top 5% of the d_{xy} distribution between North and South homozygotes. **b** Distribution of nucleotide diversity (dark gray line) of 61 Atlantic herring individuals across chromosomes 6, 12, 17 and 23 containing inversions, showing that certain inversions (e.g., chromosome 17) occur in genomic regions of elevated high nucleotide diversity.

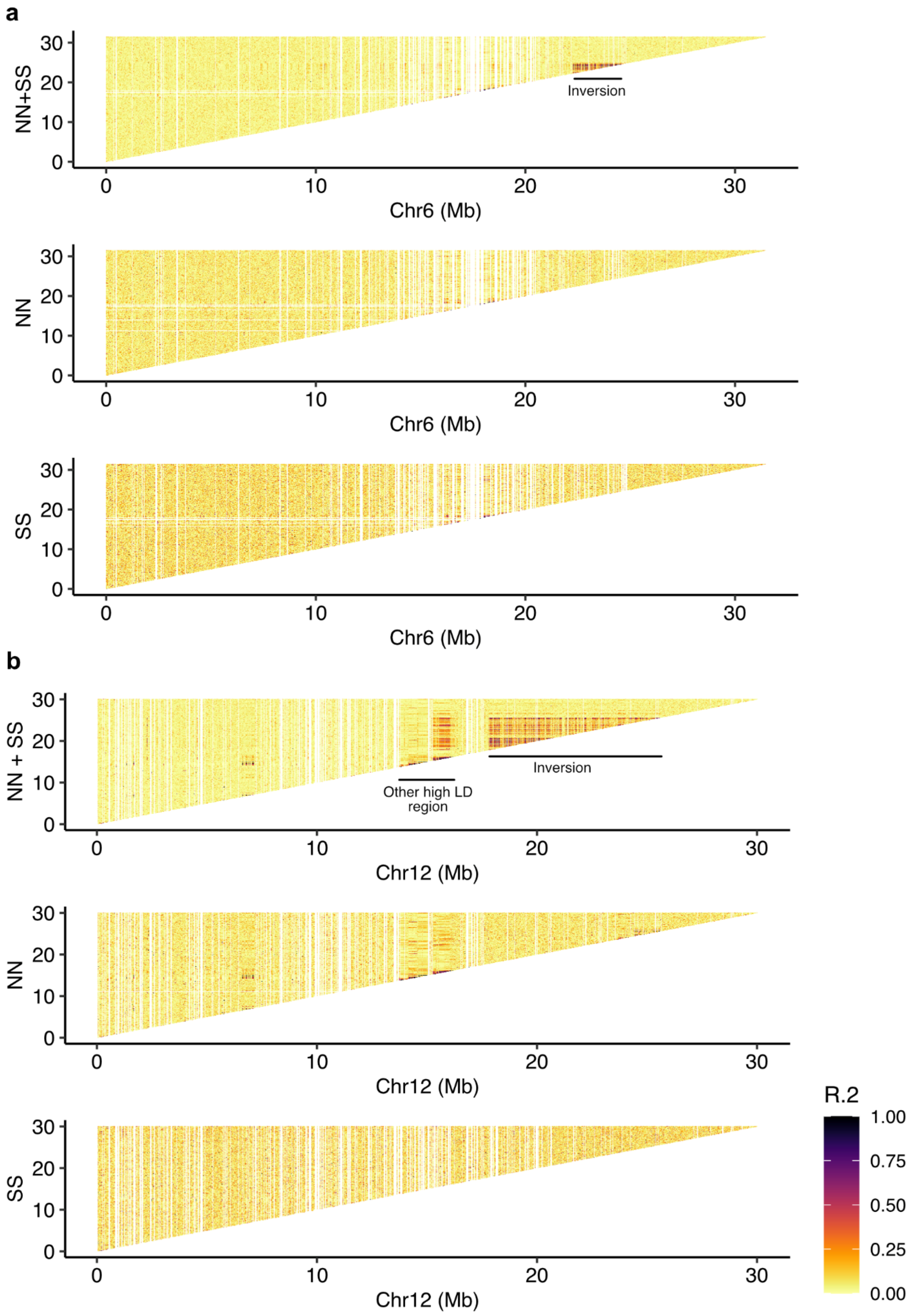

a
NN+SS
NN
SS
Inversion
Chr6 (Mb)
b
NN + SS
NN
SS
Other high LD region
Inversion
Chr12 (Mb)
R.2
1.00
0.75
0.50
0.25
0.00

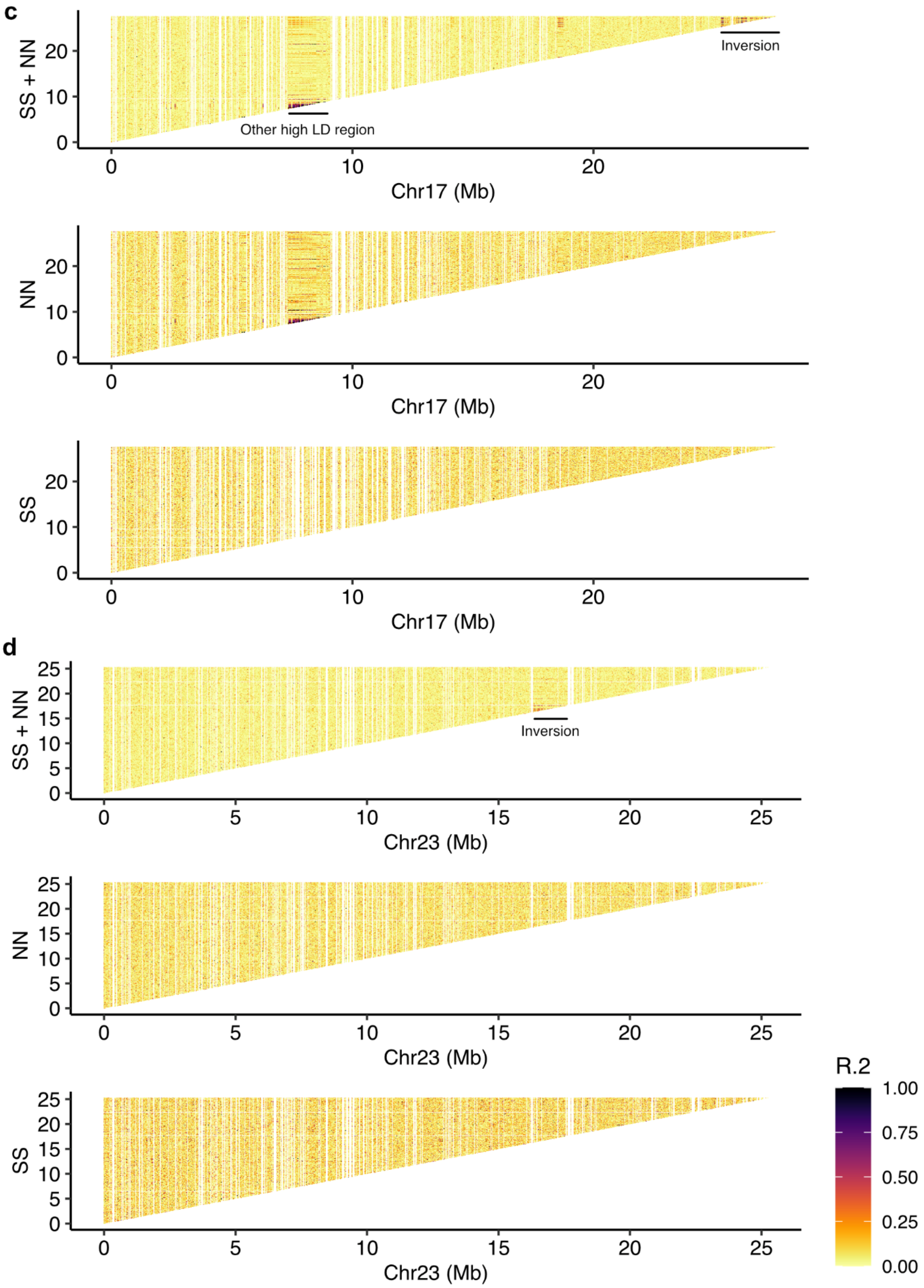
c
SS + NN
20
10
0
Inversion
Other high LD region
0
10
20
Chr17 (Mb)
NN
SS
d
SS + NN
25
15
5
Inversion
Chr23 (Mb)
R.2
1.00
0.75
0.50
0.25
0.00

Supplementary Fig. 10. Linkage disequilibrium (LD) in herring a Chr6, b Chr12, c Chr17 and d Chr23, containing inversions. For each chromosome, LD is plotted as R^2. Top plots combine Southern and Northern homozygotes (*NN*+*SS*), evidencing the high LD within the inversion regions in the context of the entire chromosome which should be recombining freely, with the exception of a few other regions of high LD. The two bottom plots only include Northern (*NN*) or Southern (*SS*) homozygotes demonstrating the lack of LD, suggesting normal recombination rate within the inversion regions. Number of North homozygotes for *N* alleles is $n_{chr6} = 20$, $n_{chr12} = 19$, $n_{chr17} = 16$ and $n_{chr23} = 16$, and number of South homozygotes for *S* alleles is $n_{chr6} = 15$, $n_{chr12} = 15$, $n_{chr17} = 15$ and $n_{chr23} = 13$.

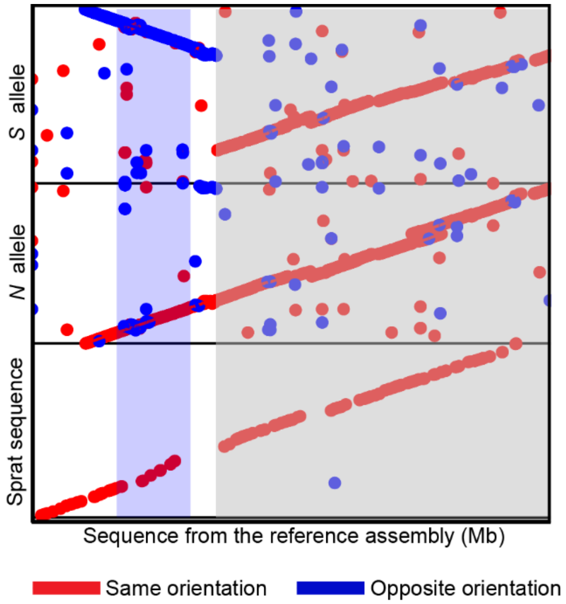

Supplementary Fig. 11. Dot plot comparing high-F_{ST} and inversion sequence on chromosome 17 from the Atlantic herring reference assembly with sequences from sprat assembly, *S* allele from PacBio assembly, and *N* allele from PacBio assembly. Red and blue colors represent forward and reverse orientations of the alignments, respectively. Blue shaded region corresponds to high-F_{ST} sequence, while grey shaded region corresponds to the inversion sequence. The sequence lengths for sprat sequence, *S* allele, and *N* allele are 2.8 Mb, 2.6 Mb, and 2.8 Mb, respectively.

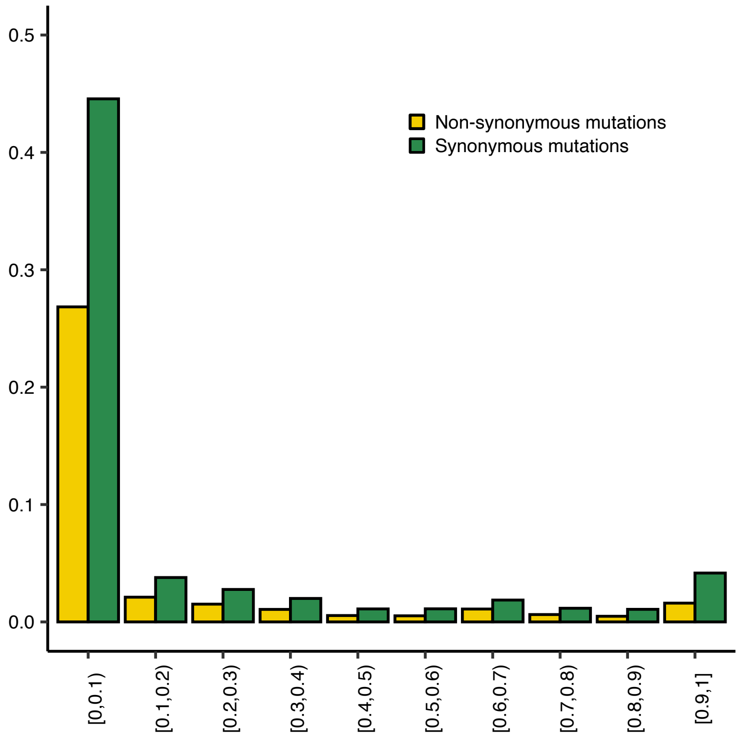

Supplementary Fig. 12. Site frequency spectra of derived non-synonymous (yellow bars) and synonymous (green bars) mutations for the whole genome. Allele frequencies were derived from 61 Atlantic herring individuals.

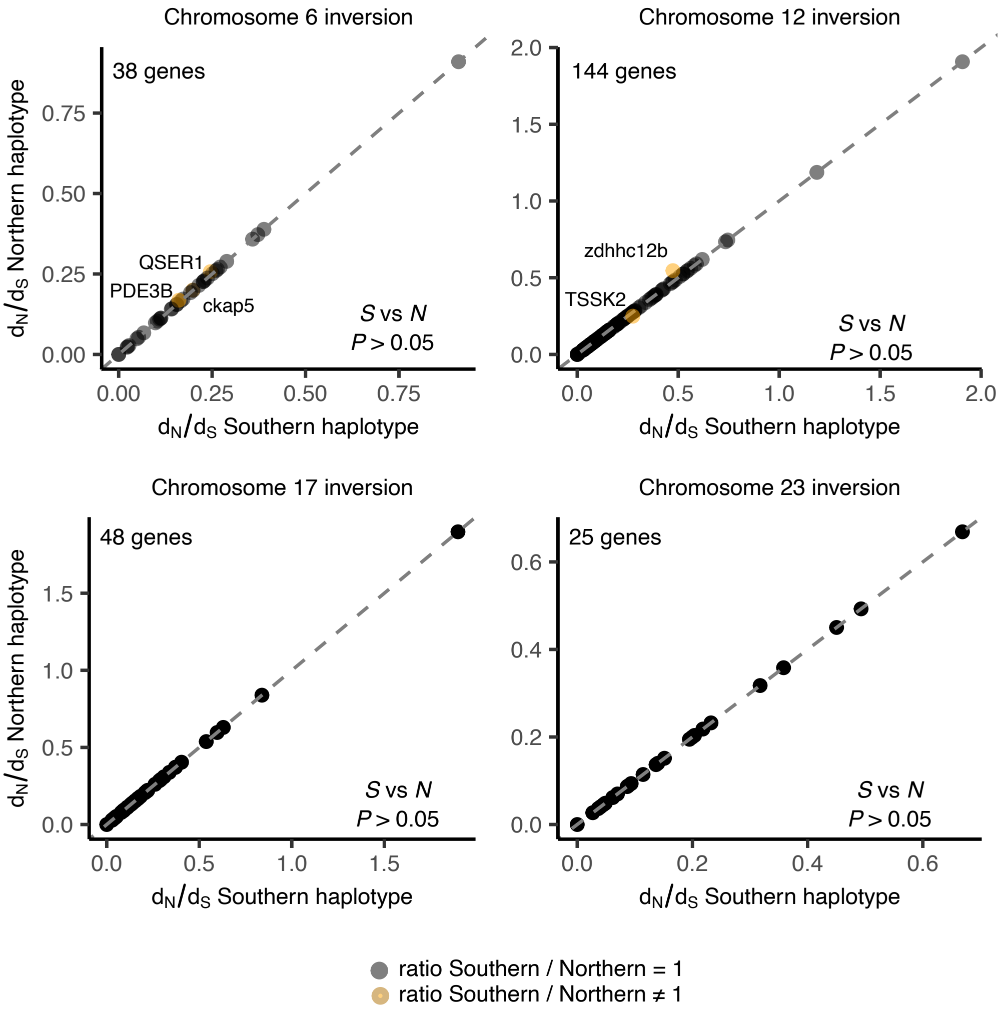

Supplementary Fig. 13. Comparison of d_N/d_S ratios for genes within the Northern (*N*) and Southern (*S*) inversion haplotypes. d_N/d_S values were calculated considering a consensus sequence for *N* and *S* haplotypes obtained from homozygotes. Genes where the ratio of d_N/d_S between *N* and *S* haplotypes deviates from one are highlighted in orange. A two-sided *t*-test reveals no significant differences in the distribution of d_N/d_S values between *N* and *S* haplotypes.

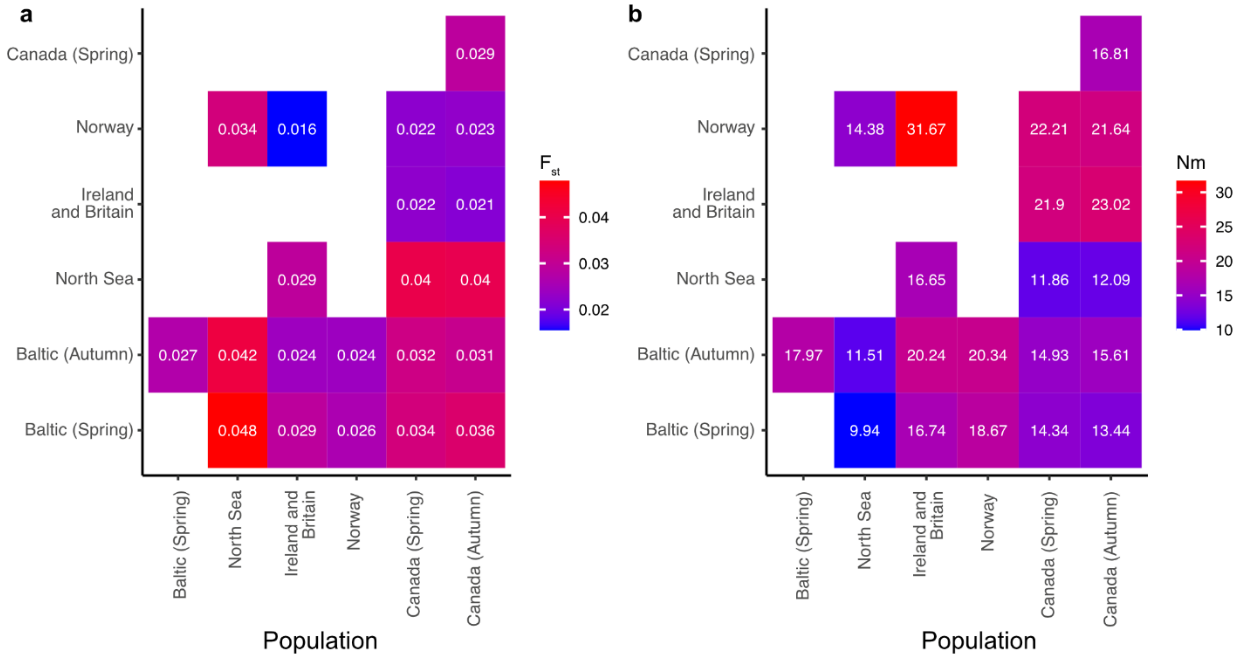

Supplementary Fig. 14. **a Differentiation as F_{st} and b population migration rate (Nm) between Atlantic herring populations.** Sample sizes are as follows: Canada Spring n = 6, Canada Autumn n = 6, Norway n = 11, Ireland and Britain n = 15, Baltic Spring n = 14, Baltic Autumn n = 6.

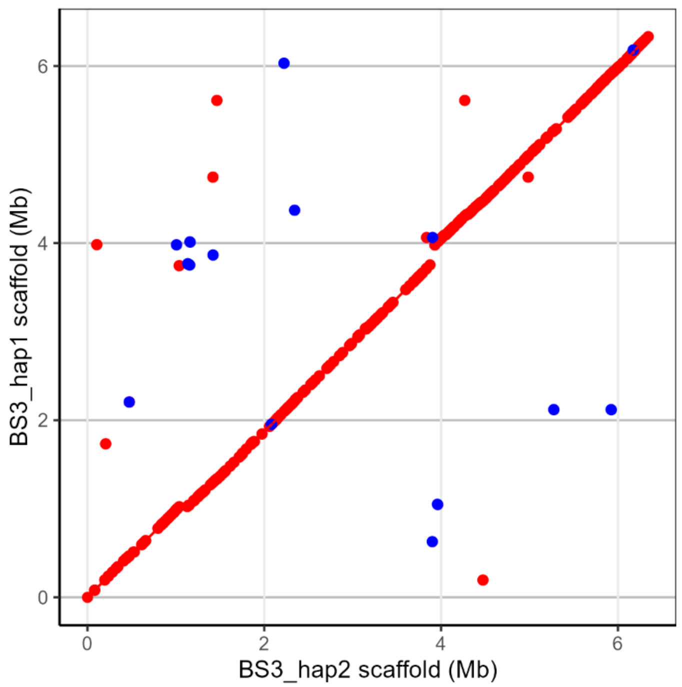

Supplementary Fig. 15. **Dot plot showing alignment of Chr6 inversion-scaffold from BS3_hap1 (Y-axis) and BS3_hap2 (X-axis).**

Supplementary Table 1. Genome statistics for Celtic Sea (CS) and Baltic Sea (BS) PacBio genome assemblies using hifiasm assembler. Hap1 and hap2 are the two haplotype genomes and their statistics are shown on the top and bottom row for each sample, respectively.

Samples	Genome size (Mb)	Genome completeness (%)	No. contigs	N50 (Kb)
CS2	769.1	91.5	4364	524.8
	744.6	91.6	3314	547.9
CS4	776.3	92.8	3253	693.1
	751.3	92.8	2539	731.1
CS5	768.1	91.6	3817	545.0
	745.9	91.6	3037	569.8
CS7	783.6	92.0	3929	469.5
	751.1	90.6	3117	512.3
CS8	781.7	91.9	4100	526.5
	743.2	91.5	3280	504.5
CS10	773.1	90.7	3997	452.8
	756.6	90.3	3228	472.4
BS1	776.2	91.6	3756	576.2
	751.8	91.4	3028	606.5
BS2	788.3	91.8	3769	540.9
	759.7	90.7	2952	539.8
BS3	779.2	92.4	3331	680.2
	758.5	92.1	2725	661.8
BS4	792.2	92.8	3309	737.1
	760.1	92.0	2570	735.4
BS5	780.3	90.6	4045	494.0
	751.8	91.1	3259	480.0
BS6	787.8	92.2	3771	572.2
	764.3	92.3	3083	557.1
European sprat	850.3	91.8	4748	544.6
	802.4	91.7	3611	590.2

Supplementary Table 2. Genome statistics for Celtic Sea (CS) and Baltic Sea (BS) PacBio genome assemblies using HiCanu assembler. Hap1 and hap2 are the two haplotype genomes and their statistics are shown on the top and bottom row for each sample, respectively.

Samples	Genome size (Mb)	No. contigs	N50 (Kb)
CS2	747.7	3497	554.3
	774.7	9155	234.1
CS4	797.7	3029	744.8
	721.9	5289	306.9
CS5	760.0	3202	564.2
	728.4	6000	267.7
CS7	759.4	3468	489.6
	710.3	5736	244.7
CS8	760.6	3347	553.2
	738.0	6330	243.4
CS10	763.6	3447	507.1
	710.8	5969	231.9
BS1	626.3	3064	626.3
	281.3	5584	281.3
BS2	586.6	3000	586.6
	265.6	5802	265.6
BS3	724.0	2682	724.0
	321.1	5559	321.1
BS4	805.2	2615	805.2
	342.4	5750	342.4
BS5	531.4	3357	531.4
	241.5	5983	241.5
BS6	606.3	3083	606.3
	270.9	5797	270.9

Supplementary Table 3. Inversion breakpoints for *S* (Celtic Sea) and *N* (Baltic Sea) haplotypes on four chromosomes based on the inversion scaffolds from the PacBio assemblies (CS10_hap1 and BS3_hap1 assemblies for chromosome 6 inversion; and CS10_hap1 and BS3_hap2 assemblies for inversions on chromosomes 12, 17, and 23).

Chromosome	Sample	Proximal breakpoint	Distal breakpoint
6	Celtic	1,144,464	3,774,578
	Baltic	1,051,601	3,747,148
12	Celtic	1,156,916	8,338,930
	Baltic	1,215,813	8,428,837
17	Celtic	741,635	2,249,708
	Baltic	687,774	2,468,994
23	Celtic	854,555	2,134,906
	Baltic	788,123	1,676,584

Supplementary Table 4. Sequence analysis of inverted duplications flanking the inversion alleles

	S_allele: proximal vs. distal	*N_allele*: proximal vs. distal	proximal: *N allele* vs. *S allele*	distal: *N allele* vs. *S allele*
chromosome 6				
length (bp)	8346 vs. 7651	21931 vs. 21641	21931 vs. 8346	21641 vs. 7651
% identity	94.48	99.00	96.97	94.21
query coverage	91	99	90	99
chromosome 12				
length (bp)	6858 vs. 6874	7811 vs. 7834	7811 vs. 6858	7834 vs. 6874
% identity	98.62	98.61	99.39	99.07
query coverage	99	99	99	100
chromosome 17				
length (bp)		7865 vs. 7813		
% identity		95.80		
query coverage		99		

Supplementary Table 5. Structural variations surrounding inversion breakpoints.

Inversion	Location	Type of SV	Description	Gene
Chromosome 6	Proximal and distal	Inverted duplication of 35 kb in S alleles and 45 kb in N alleles	In the reference, co-ordinates are - chr6:22223000-22261500 and chr6:24913000-24951000	*BTLN-2*
	Proximal	30-50 kb insertion in CS10_hap1, CS10_hap2, BS2_hap2, BS3_hap2, BS6_hap1		
	Distal	30-200 kb divergent sequence. 200 kb sequence is present in BS3_hap1, BS5_hap2, and BS6_hap2		
Chromosome 12	Proximal and distal	Inverted duplication of 8 kb in both S and N alleles	In the reference, co-ordinates are chr12:17818247-17826319 and chr12:25602941-25610712	*FUT-9*
	Distal	30 kb palindromic sequence		
Chromosome 17	Proximal and distal	Inverted duplication of 60 kb in both S and N alleles	In the reference, co-ordinates are chr17:25728061-25805444	NA
	Proximal	20 kb sequence in one-three copies in all S and N alleles		fatty acid binding protein, liver-type-like
	Distal	50 kb palindromic sequence in all S and N alleles except BS1_hap1, BS3_hap1, BS4_hap2, BS6_hap2. These exception alleles also don't have a telomeric sequence outside inversion. BS5_hap2 has one more palindromic sequence. It is absent in the reference assembly.	One of the copies is very fragmented. Has many repeats.	NA

		The telomeric sequence next to the breakpoint is of variable length in all assemblies, ranging from 0 kb to 300 kb		
Chromosome 23	Proximal	130 kb sequence is present in 3-4 copies in all S and N alleles	Additional copy of this sequence is present in the opposite orientation in all N alleles, except BS4_hap2. It is absent in all S alleles except CS2_hap2 and CS10_hap1.	NA
		13 kb insertion in eight N alleles; BS2_hap1, BS2_hap2, BS3_hap1, BS3_hap2, BS4_hap1, BS4_hap2, BS5_hap1, BS6_hap2.		NACHT, LRR and PYD domains-containing protein 12-like
	Distal	20 kb sequence in two copies. Only 10 kb of it is present in all S and N alleles. The entire 20 kb is present in CS7_hap2, CS8_hap1, and BS2_hap1<br>Insertion of variable lengths ranging from 50 to 700 kb. (CS2_hap2 at proximal, 300 kb<br>CS5_hap1 at distal, 50 kb; CS5_hap2 at distal, 100 kb; CS7_hap1 and hap2 at distal, 700 kb; CS8_hap1 at distal, 200 kb; CS8_hap2 at distal, 300 kb, 150 kb of it is homologous to that of CS8_hap1; CS10_hap1 at distal, 250 kb; BS1_hap1 at distal, around 250 kb, fragmented BS3_hap1 at distal, 100 kb; BS5_hap1 at distal, 500 kb)		nuclear body protein SP140-like protein

Supplementary Table 6. χ^2 test (d.f. = 1) of possible enrichment of non-synonymous mutations among extremely differentiated SNPs (dAF > 0.95)

Inversion	Observed	Expected	*P*-value (d.f. = 1)
Chromosome 6	7	5	0.37
Chromosome 12	13	9	0.18
Chromosome 17	2	2	1.00
Chromosome 23	3	2	0.47

Supplementary Table 7. Test of Hardy-Weinberg equilibrium for Atlantic herring chromosomal inversions in different populations. Observed genotype frequencies were obtained from Fig. 1. Highlighted rows indicate fixation of one of the alleles and the fixed alleles are indicated in bold. Two-sided χ^2 was used.

Population	f(*SS*)	f(*NS*)	f(*NN*)	Sample size	f(*N*)	χ^2 (df = 1)
			Chromosome 6			
Canada Atlantic Spring	0.000	0.000	**1.000**	6	1.000	
Canada Atlantic Autumn	0.000	0.000	**1.000**	6	1.000	
Norway Atlantic Spring	0.000	0.091	0.909	11	0.955	0.002
Baltic Spring	0.000	0.000	**1.000**	14	1.000	
Baltic Autumn	0.000	0.000	**1.000**	6	1.000	
North Sea	0.333	0.667	0.000	3	0.333	0.250
Ireland and Britain	**1.000**	0.000	0.000	15	0.000	
			Chromosome 12			
Canada Atlantic Spring	0.000	0.000	**1.000**	6	1.000	
Canada Atlantic Autumn	0.167	0.167	0.667	6	0.750	0.309
Norway Atlantic Spring	0.000	0.273	0.727	11	0.864	0.025
Baltic Spring	0.000	0.000	**1.000**	14	1.000	
Baltic Autumn	0.167	0.000	0.833	6	0.833	1.000
North Sea	**1.000**	0.000	0.000	3	0.000	
Ireland and Britain	0.933	0.067	0.000	15	0.033	0.001
			Chromosome 17			
Canada Atlantic Spring	0.000	0.000	**1.000**	6	1.000	
Canada Atlantic Autumn	0.000	0.000	**1.000**	6	1.000	
Norway Atlantic Spring	0.000	0.545	0.455	11	0.727	0.141
Baltic Spring	0.000	0.071	0.929	14	0.964	0.001
Baltic Autumn	0.000	0.500	0.500	6	0.750	0.111
North Sea	0.333	0.667	0.000	3	0.333	0.250
Ireland and Britain	**1.000**	0.000	0.000	15	0.000	
			Chromosome 23			
Canada Atlantic Spring	0.000	0.333	0.667	6	0.833	0.040
Canada Atlantic Autumn	0.000	0.000	**1.000**	6	1.000	
Norway Atlantic Spring	0.091	0.091	0.818	11	0.864	0.377
Baltic Spring	0.000	0.286	0.714	14	0.857	0.028

Baltic Autumn	0.000	0.000	**1.000**	6	1.000	
North Sea	0.333	0.333	0.333	3	0.500	0.111
Ireland and Britain	0.867	0.133	0.000	15	0.067	0.005

Supplementary Table 8. Genome statistics of hybrid scaffold assemblies based on Bionano analysis. Hap1 and hap2 are the two haplotype genomes and their statistics are shown on the top and bottom row for each sample, respectively. These hybrid scaffolds included large number of Ns.

Samples	Genome size (Mb)	No. contigs	N50 (Mb)	N's per 100 Kb (Kb)	Not scaffolded sequence (Mb)
CS10	937.3	123	23.0	28.6	104.2
	950.5	129	22.9	29.3	85.0
BS3	866.7	129	23.5	19.9	85.1
	871.4	135	23.4	21.0	70.0

Supplementary Table 9. Genotypes of 35 individuals from Southern (*S*) and Northern (*N*) haplotypes at each inversion, determined by local PCA and diagnostic SNPs for each inversion.

Individual	Population	Inversion			
		Chr6	Chr12	Chr17	Chr23
AAL1_CelticSea_Atlantic_Winter	Celtic Sea	*SS*	*NS*	*SS*	*NS*
AAL2_CelticSea_Atlantic_Winter	Celtic Sea	*SS*	*SS*	*SS*	*NS*
AAL3_Celticsea_Atlantic_Winter	Celtic Sea	*SS*	*SS*	*SS*	*SS*
CS10	Celtic Sea	*SS*	*SS*	*SS*	*SS*
CS4	Celtic Sea	*SS*	*SS*	*SS*	*SS*
CS5	Celtic Sea	*SS*	*SS*	*SS*	*SS*
CS7	Celtic Sea	*SS*	*SS*	*SS*	*SS*
CS8	Celtic Sea	*SS*	*SS*	*SS*	*SS*
CS2	Celtic Sea	*SS*	*SS*	*SS*	*SS*
AK1_Downs_Atlantic_Winter	English Channel	*SS*	*SS*	*SS*	*SS*
AK2_Downs_Atlantic_Winter	English Channel	*SS*	*SS*	*SS*	*SS*
AK3_Downs_Atlantic_Winter	English Channel	*SS*	*SS*	*SS*	*SS*
Z12_IsleofMan_Atlantic_Autumn	Irish Sea	*SS*	*SS*	*SS*	*SS*
Z14_IsleofMan_Atlantic_Autumn	Irish Sea	*SS*	*SS*	*SS*	*SS*
Z4_IsleofMan_Atlantic_Autumn	Irish Sea	*SS*	*SS*	*SS*	*SS*
BS1	Baltic Sea	*NN*	*NN*	*NN*	*NN*
BS2	Baltic Sea	*NN*	*NN*	*NN*	*NS*
BS3	Baltic Sea	*NN*	*NN*	*NN*	*NN*
BS4	Baltic Sea	*NN*	*NN*	*NN*	*NN*
BS5	Baltic Sea	*NN*	*NN*	*NS*	*NN*
BS6	Baltic Sea	*NN*	*NN*	*NN*	*NN*
Fehmarn3_Fehmarn_Baltic_Autumn	Baltic Sea	*NN*	*NN*	*NS*	*NN*
Fehmarn44_Fehmarn_Baltic_Autumn	Baltic Sea	*NN*	*NN*	*NS*	*NN*
Fehmarn6_Fehmarn_Baltic_Autumn	Baltic Sea	*NN*	*NN*	*NS*	*NN*
Gavle100_Gävle_Baltic_Autumn	Baltic Sea	*NN*	*NN*	*NN*	*NN*
Gavle54_Gävle_Baltic_Autumn	Baltic Sea	*NN*	*SS*	*NN*	*NN*
Gavle98_Gävle_Baltic_Autumn	Baltic Sea	*NN*	*NN*	*NN*	*NN*
BF16_Hästskär_Baltic_Spring	Baltic Sea	*NN*	*NN*	*NN*	*NN*
BF18_Hästskär_Baltic_Spring	Baltic Sea	*NN*	*NN*	*NN*	*NS*
BF19_Hästskär_Baltic_Spring	Baltic Sea	*NN*	*NN*	*NN*	*NN*
BF21_Hästskär_Baltic_Spring	Baltic Sea	*NN*	*NN*	*NN*	*NN*
BM14_Hästskär_Baltic_Spring	Baltic Sea	*NN*	*NN*	*NN*	*NN*
BM15_Hästskär_Baltic_Spring	Baltic Sea	*NN*	*NN*	*NN*	*NS*
BM16_Hästskär_Baltic_Spring	Baltic Sea	*NN*	*NN*	*NN*	*NN*
BM19_Hästskär_Baltic_Spring	Baltic Sea	*NN*	*NN*	*NN*	*NS*
