# Supplementary material for: The origin and maintenance of supergenes contributing to ecological adaptation in Atlantic herring
Source: Nat Commun. 2024 Oct 23;15:9136. doi: 10.1038/s41467-024-53079-7 (PMC11499932; doi:10.1038/s41467-024-53079-7)
Supplement: Supplementary file 3 — Reporting Summary [file 41467_2024_53079_MOESM3_ESM.pdf]

nature portfolio

Corresponding author(s): Leif Andersson

Last updated by author(s): Aug 1, 2024

Reporting Summary

Nature Portfolio wishes to improve the reproducibility of the work that we publish. This form provides structure for consistency and transparency in reporting. For further information on Nature Portfolio policies, see our Editorial Policies and the Editorial Policy Checklist.

Statistics

For all statistical analyses, confirm that the following items are present in the figure legend, table legend, main text, or Methods section.

n/a	Confirmed	
☐	☒	The exact sample size (*n*) for each experimental group/condition, given as a discrete number and unit of measurement
☐	☒	A statement on whether measurements were taken from distinct samples or whether the same sample was measured repeatedly
☐	☒	The statistical test(s) used AND whether they are one- or two-sided<br>*Only common tests should be described solely by name; describe more complex techniques in the Methods section.*
☒	☐	A description of all covariates tested
☐	☒	A description of any assumptions or corrections, such as tests of normality and adjustment for multiple comparisons
☐	☒	A full description of the statistical parameters including central tendency (e.g. means) or other basic estimates (e.g. regression coefficient) AND variation (e.g. standard deviation) or associated estimates of uncertainty (e.g. confidence intervals)
☐	☒	For null hypothesis testing, the test statistic (e.g. *F*, *t*, *r*) with confidence intervals, effect sizes, degrees of freedom and *P* value noted<br>*Give P values as exact values whenever suitable.*
☒	☐	For Bayesian analysis, information on the choice of priors and Markov chain Monte Carlo settings
☒	☐	For hierarchical and complex designs, identification of the appropriate level for tests and full reporting of outcomes
☒	☐	Estimates of effect sizes (e.g. Cohen's *d*, Pearson's *r*), indicating how they were calculated

Our web collection on statistics for biologists contains articles on many of the points above.

Software and code

Policy information about availability of computer code

Data collection	Short-read whole genome sequencing data was generated through Illumina HiSeq and HiFi long-read whole genome sequencing data was generated through PacBio Sequel II.
Data analysis	Genome assembly construction: HiCanu (v2.0), Purge_dups and hifiasm (v0.16.1-r375), QUAST (v5.0.2), BUSCO (v5.beta), hybridScaffol pipeline (Bionano Solve 3.7).<br>Long-read alignments: minimap2 (v2.22-r1101), MUMmer (v4.0.0rc1), BLAST (v2.11.0+).<br>Genome graph construction: pggb (v0.3.1), odgi (v0.7.3).<br>Short-read alignment and variant calling: FastQC (0.11.9), BWA-MEM (v.0.7.17), Picard (v2.10.3), Sentieon wrapper (release 201911), GATK (as implemented in Sentieon).<br>Generating consensus sequences: bedtools (v.2.29.2), bcftools (v.1.12), vcftools(v.0.1.16), samtools (v.1.12), satsuma2 (v.2016-12-07), Biostrings (v.2.68.1), biomaRt (v.2.56.1), GenomicRanges (v1.52.0) and tidyverse (v.2.0.0), mafft (v7.407), AMAS summary.<br>Population genomic analyses: bcftools (v.1.12), vcftools, tidyverse, ggplot2 (3.4.2) and ggrstar (1.0.1), lstruct (October 2022), pixy (v1.2.5)<br>Phylogenetics: IQ-TREE (v. 2.0-rc2), FigTree (v.1.4.4).<br>Mutation load analysis: agat (v.0.8.0), bedtools, R 4.3.0, SNPEff (v5.1), bcftools, vcftools, RepeatModeler (2.0.1), BLAST (v.2.11.0+), TEclassTest (v.2.1.3c), biopython (v.1.79), RepeatMasker (2.0.1), CDHit (v.4.8.1)<br>Allele-sharing detection: vcftools, SnpEff (v3.4), R 4.3.0.<br>The analyses of data have been carried out with publicly available software and all are cited in the Methods section. Custom scripts are avaialble in https://zenodo.org/records/12786351 and https://zenodo.org/records/12792460.

For manuscripts utilizing custom algorithms or software that are central to the research but not yet described in published literature, software must be made available to editors and reviewers. We strongly encourage code deposition in a community repository (e.g. GitHub). See the Nature Portfolio guidelines for submitting code & software for further information.

Data

Policy information about availability of data

All manuscripts must include a data availability statement. This statement should provide the following information, where applicable:

- Accession codes, unique identifiers, or web links for publicly available datasets
- A description of any restrictions on data availability
- For clinical datasets or third party data, please ensure that the statement adheres to our policy

The sequence data generated in this study will be available from BioProject PRJNA1023520. This study also used previously available data from BioProject PRJNA642736. The European sprat PacBio reads are deposited in PRJNA1023385 from another publication. The European sprat assembly generated in this work is deposited in figshare (https://doi.org/10.6084/m9.figshare.24354943). Other relevant input and output files are similarly deposited in figshare which will be public upon publication.

Research involving human participants, their data, or biological material

Policy information about studies with human participants or human data. See also policy information about sex, gender (identity/presentation), and sexual orientation and race, ethnicity and racism.

Reporting on sex and gender	*Use the terms sex (biological attribute) and gender (shaped by social and cultural circumstances) carefully in order to avoid confusing both terms. Indicate if findings apply to only one sex or gender; describe whether sex and gender were considered in study design; whether sex and/or gender was determined based on self-reporting or assigned and methods used.*<br>*Provide in the source data disaggregated sex and gender data, where this information has been collected, and if consent has been obtained for sharing of individual-level data; provide overall numbers in this Reporting Summary. Please state if this information has not been collected.*<br>*Report sex- and gender-based analyses where performed, justify reasons for lack of sex- and gender-based analysis.*
Reporting on race, ethnicity, or other socially relevant groupings	*Please specify the socially constructed or socially relevant categorization variable(s) used in your manuscript and explain why they were used. Please note that such variables should not be used as proxies for other socially constructed/relevant variables (for example, race or ethnicity should not be used as a proxy for socioeconomic status).*<br>*Provide clear definitions of the relevant terms used, how they were provided (by the participants/respondents, the researchers, or third parties), and the method(s) used to classify people into the different categories (e.g. self-report, census or administrative data, social media data, etc.)*<br>*Please provide details about how you controlled for confounding variables in your analyses.*
Population characteristics	*Describe the covariate-relevant population characteristics of the human research participants (e.g. age, genotypic information, past and current diagnosis and treatment categories). If you filled out the behavioural & social sciences study design questions and have nothing to add here, write "See above."*
Recruitment	*Describe how participants were recruited. Outline any potential self-selection bias or other biases that may be present and how these are likely to impact results.*
Ethics oversight	*Identify the organization(s) that approved the study protocol.*

Note that full information on the approval of the study protocol must also be provided in the manuscript.

Field-specific reporting

Please select the one below that is the best fit for your research. If you are not sure, read the appropriate sections before making your selection.

☐ Life sciences ☐ Behavioural & social sciences ☒ Ecological, evolutionary & environmental sciences

For a reference copy of the document with all sections, see nature.com/documents/nr-reporting-summary-flat.pdf

Ecological, evolutionary & environmental sciences study design

All studies must disclose on these points even when the disclosure is negative.

Study description	Long-read PacBio HiFi data for 12 Atlantic herring, representing two inversion haplotypes was used to confirm inversions and characterize breakpoint regions. Long-read PacBio HiFi data for one European sprat was used to find the ancestral state of the inversion and as an outgroup species for evolutionary analyses. Short-read Illumina data from the same 12 samples along with additional 49 Atlantic herring and 30 Pacific herring was used in various evolutionary analyses.
Research sample	The study was carried out on 61 Atlantic herring (Clupea harengus), collected from waters near Ireland and Britain in Northeast Atlantic Ocean and Baltic Sea. In addition, we used 30 Pacific herring (Clupea pallasii) samples collected from Vancouver, Strait of Georgia. One outgroup species of European sprat (Sprattus sprattus) was collected from Langenuen in the outer part of Hardangerfjorden, Norway (59.975°N, 5.376°E).

Sampling strategy	This paper is built on the findings from a bigger project that compared Atlantic herring populations across its species distribution that compared seven different populations to each other. In the comparison "Atlantic herring from waters around Ireland and Britain vs. other parts of the Northeast Atlantic Ocean", four of the signals were for inversions that showed allele frequency cline from north to south Atlantic Ocean. In this study, we sampled six individuals from north (Baltic Sea) and six individuals from south (Celtic Sea) to confirm the inversions and further explore their evolution, with the assumption that these two sets of samples are fixed for either of the inversion haplotype. We also sampled one European sprat which was used as an outgroup species to Atlantic herring. We combined this dataset with previously sequenced samples from these two regions, that included 49 Atlantic herring and 30 Pacific herring.
Data collection	We generated Illumina 2 x 150 bp short read and PacBio HiFi whole genome sequencing data for 12 Atlantic herring samples from Texas A&M Institute for Genome Sciences and Society and Institute of Genome Sciences of University of Maryland School of Medicine, respectively. We combined this dataset with 49 Atlantic herring and 30 Pacific herring samples that previously sequenced using Illumina 2 x 150 bp short read at SciLifeLab of Uppsala university. Whole genome of one European sprat sample was sequenced using PacBio HiFi at SciLifeLab of Uppsala university.
Timing and spatial scale	Atlantic herring samples were collected between 1979 to 2020 from the Northeast and Northwest Atlantic Ocean and Baltic Sea. European sprat sample was collected in 2021.
Data exclusions	No data were excluded for any analysis.
Reproducibility	All results are reproducible. All the analysis pipeline required to reproduce the results are described in the methods section.
Randomization	Samples were allocated into groups based on their sampling locations.
Blinding	*Describe the extent of blinding used during data acquisition and analysis. If blinding was not possible, describe why OR explain why blinding was not relevant to your study.*

Did the study involve field work? ☐ Yes ☒ No

Reporting for specific materials, systems and methods

We require information from authors about some types of materials, experimental systems and methods used in many studies. Here, indicate whether each material, system or method listed is relevant to your study. If you are not sure if a list item applies to your research, read the appropriate section before selecting a response.

Materials & experimental systems

n/a	Involved in the study
☒	☐ Antibodies
☒	☐ Eukaryotic cell lines
☒	☐ Palaeontology and archaeology
☐	☒ Animals and other organisms
☒	☐ Clinical data
☒	☐ Dual use research of concern
☒	☐ Plants

Methods

n/a	Involved in the study
☒	☐ ChIP-seq
☒	☐ Flow cytometry
☒	☐ MRI-based neuroimaging

Animals and other research organisms

Policy information about studies involving animals; ARRIVE guidelines recommended for reporting animal research, and Sex and Gender in Research

Laboratory animals	This study did not involve any laboratory animals
Wild animals	Fish samples were captured by fishermen under supervision. All samples were over two years of age. The sex was recorded after sampling. The animal was killed in humanly manner and tissues were extracted on-site. The tissues were then flash frozen in liquid nitrogen and carried to the laboratory.
Reporting on sex	This study is independent on sex but the 12 samples used in this study were all males because it was difficult to obtain high molecular weight DNA required for long read sequencing from the tissues other than testes.
Field-collected samples	*For laboratory work with field-collected samples, describe all relevant parameters such as housing, maintenance, temperature, photoperiod and end-of-experiment protocol OR state that the study did not involve samples collected from the field.*
Ethics oversight	*Identify the organization(s) that approved or provided guidance on the study protocol, OR state that no ethical approval or guidance was required and explain why not.*

Note that full information on the approval of the study protocol must also be provided in the manuscript.

Plants

Seed stocks	*Report on the source of all seed stocks or other plant material used. If applicable, state the seed stock centre and catalogue number. If plant specimens were collected from the field, describe the collection location, date and sampling procedures.*
Novel plant genotypes	*Describe the methods by which all novel plant genotypes were produced. This includes those generated by transgenic approaches, gene editing, chemical/radiation-based mutagenesis and hybridization. For transgenic lines, describe the transformation method, the number of independent lines analyzed and the generation upon which experiments were performed. For gene-edited lines, describe the editor used, the endogenous sequence targeted for editing, the targeting guide RNA sequence (if applicable) and how the editor was applied.*
Authentication	*Describe any authentication procedures for each seed stock used or novel genotype generated. Describe any experiments used to assess the effect of a mutation and, where applicable, how potential secondary effects (e.g. second site T-DNA insertions, mosiacism, off-target gene editing) were examined.*
