# Supplementary material for: The origin and maintenance of supergenes contributing to ecological adaptation in Atlantic herring
Source: Nat Commun. 2024 Oct 23;15:9136. doi: 10.1038/s41467-024-53079-7 (PMC11499932; doi:10.1038/s41467-024-53079-7)
Supplement: Supplementary file 4 — Description of Additional Supplementary Files [file 41467_2024_53079_MOESM4_ESM.pdf]

Description of Additional Supplementary Files

Supplementary Data 1. Genes within the inverted region and within 200 kb flanking the inversion breakpoints (shaded grey) for inversions on chromosomes 6, 12, 17, and 23 in Atlantic herring.

Supplementary Data 2. Information on non-synonymous SNPs showing extreme genetic differentiation between inversion haplotypes (dAF > 0.95) in the inversion regions.
